# Identification of NF-κB as Determinant of Posttraumatic Stress Disorder and Its Inhibition by the Chinese Herbal Remedy *Free and Easy Wanderer*

**DOI:** 10.3389/fphar.2017.00181

**Published:** 2017-04-06

**Authors:** Chunlan Hong, Anja Schüffler, Ulrich Kauhl, Jingming Cao, Ching-Fen Wu, Till Opatz, Eckhard Thines, Thomas Efferth

**Affiliations:** ^1^Department of Pharmaceutical Biology, Institute of Pharmacy and Biochemistry, Johannes Gutenberg UniversityMainz, Germany; ^2^Institut für Biotechnologie und Wirkstoff Forschung gGmbHKaiserslautern, Germany; ^3^Institute of Molecular Physiology, Johannes Gutenberg UniversityMainz, Germany; ^4^Institute of Organic Chemistry, Johannes Gutenberg UniversityMainz, Germany

**Keywords:** *free and easy wanderer*, inflammation, NF-κB, pharmacognosy, posttraumatic stress disorder

## Abstract

Posttraumatic stress disorder (PTSD) is a mental disorder developing after exposure to traumatic events. Although psychotherapy reveals some therapeutic effectiveness, clinically sustainable cure is still uncertain. Some Chinese herbal formulae are reported to work well clinically against mental diseases in Asian countries, but the safety and their mode of action are still unclear. In this study, we investigated the mechanisms of Chinese remedy *free and easy wanderer* (FAEW) on PTSD. We used a reverse pharmacology approach combining clinical data to search for mechanisms of PTSD with subsequent *in vitro* verification and bioinformatics techniques as follows: (1) by analyzing microarray-based transcriptome-wide mRNA expression profiling of PTSD patients; (2) by investigating the effect of FAEW and the antidepressant control drug fluoxetine on the transcription factor NF-κB using reporter cell assays and western blotting; (3) by performing molecular docking and literature data mining based on phytochemical constituents of FAEW. The results suggest an involvement of inflammatory processes mediated through NF-κB in the progression of PTSD. FAEW was non-cytotoxic *in vitro* and inhibited NF-κB activity and p65 protein expression. FAEW's anti-inflammatory compounds, i.e., paeoniflorin, isoliquiritin, isoliquiritin apioside and ononin were evaluated for binding to IκK and p65-RelA in a molecular docking approach. Paeoniflorin, albiflorin, baicalin, isoliquiritin and liquiritin have been reported to relieve depression *in vivo* or in clinical trials, which might be the active ingredients for FAEW against PTSD.

## Introduction

During the past decades, the developments in science and technology have driven medical research further toward “disease-based” approaches, which may be critical for some chronical mental diseases, since no significant morphological changes can be observed, while patients are suffering for a long time. In 1977, a bio-psycho-social medical theory was postulated by the psychiatrist George L. Engel at the University of Rochester (White, 2005). He suggested holistic approaches to handle medical issues, because health is a comprising status of physical, mental and social well-being and not merely the absence of diseases or infirmity, indicating the new direction to look for new approaches from complementary medicine.

With a specific philosophical background and treatment principles, traditional Chinese medicine (TCM) emphasizes the balance of *yin* and *yang* to achieve harmony between body, mind and soul. With thousands of years of clinical practice and written documentation, TCM is especially well suited to treat difficult and complex diseases. The success should be partly attributed to diverse medicinal plants, which have been a valuable source of therapeutic agents in the treatment of cancer, neurodegenerative diseases and malaria (Houghton and Howes, 2005; Kong and Tan, 2015; Khandelwal et al., 2016). The enormous structural and chemical diversity of natural products are evolutionarily optimized for serving different biological functions (Mishra and Tiwari, 2011). In addition, the traditional knowledge on plants and well documented ethno-pharmacological information can be taken as reliable hints for further pharmacological research.

*Free and Easy Wanderer* (FAEW), is a Chinese herb formula, used in China for hundreds of years in the treatment of mood disorders, and *in vivo* results proved to reverse anxiety-like behavior and cognitive impairments after stress exposure (Wang et al., 2009). To explain how herbal formulae work in human body against mental diseases, some challenges should be discussed:

1. Mental diseases appear to us with dissociation symptoms and loss of capabilities, such as the interruption of the normally integrative functions of consciousness, memory, the identity, or perception of the environment. There are no significant pathology and morphological changes, which indicates that it may be difficult to identify suitable cellular targets for treatment. The current research mainly focuses on the investigation between neural circuits on fear conditioning and extinction (Rey et al., 2014), epigenetic changes on the hypothalamus-pituitary (HPA) axis (Vukojevic et al., 2014) and various genetic polymorphisms, e.g., *FKBP5, 5-HTTLPR, DRD2* (Kolassa et al., 2010; Walsh et al., 2014; Wilker et al., 2014). Until now, no specific pathogenic mechanism has been identified, although some progress was achieved. To solve this problem, genomic analyses of clinical patients may be one potential approach to gain insight into the pathophysiology of such diseases.
2. Chinese herbal formulae are poly-herbal preparations, frequently consisting of dozens to hundreds of chemical compounds, which might increase safety risks and hamper the agreement on their clinical usage, especially in western countries. To understand how they work in the human body, the molecular mechanisms should be clarified and possible active compounds have to be identified for further drug development.

In this paper, we first analyzed microarray-based transcriptome-wide mRNA expression profiles of patients suffering from posttraumatic stress disorder (PTSD). Based on gene network analyses and associated binding motifs of transcription factors in gene promoters, we hypothesized that inflammatory process may represent a major cause of PTSD. We investigated the cytotoxicity of FAEW and compared its effect with the antidepressant drug fluoxetine on inflammation by assessing the activity of NF-κB and the protein expression of p65. Furthermore, we identified the active compounds of FAEW by molecular docking *in silico*. Finally, we compared our results with reports in the literature and confirmed some pharmaceutical activities of the compounds.

## Materials and methods

### Gene expression profiling and network analysis of PTSD patients

Gene expression profiling from PTSD patients were searched from Pubmed, GEO dataset and Google Scholar database with the key word “PTSD” and “gene expression profiling.” Among all the results, only four were related to our analysis with data available (Segman et al., 2005; Su et al., 2008; Neylan et al., 2011; Tylee et al., 2015). Among the datasets we have chosen for our study, three were derived from blood samples (Segman et al., 2005; Neylan et al., 2011; Tylee et al., 2015), and one was originated from *post mortem* collected brain tissue biopsies (Su et al., 2008). To allow comparisons between the four datasets, consistent fold-change ratios were calculated between the control and PTSD groups. Gene symbols (or IDs) and fold-change values were uploaded into the Ingenuity Pathway Analysis (IPA) software (Jimenez-Marin et al., 2009) to determine canonical signal transduction pathways, gene functions and signaling networks predicted by dysregulated gene expressions.

### Binding motif search for transcription profiles in gene promoter sequences

Transcription factor binding site analyses were performed by the Cistrome analysis software (Liu et al., 2011). Briefly, regulated genes were input and BED formats, a tab-delimited text file defining data lines displayed in an annotation track, were retrieved with an upstream setting (promoter region) at 2 kb (Karolchik et al., 2014). SeqPos motif analyses were used to screen for enriched motifs in given regions (http://cistrome.org). Using SeqPos, we scanned all the motifs not only in Transfac, JASPAR, UniPROBE (pbm), hPDI database, but also attempted to identify *de novo* motifs using MDscan algorithm. The output of genes was ranked by −10×log(*p*-value).

### Chemicals and extracts

The FAEW extract was prepared from commercial pills (*Xiaoyao wan*) purchased from *Wanxi* Pharmaceutical Company (Henan Province, China). They were dissolved in H_2_O: MeOH: DCM in a ratio of 1: 4: 5 for 3 days. A rotary evaporator was used to remove the solvents and the final extracts were stored at −20°C. Fluoxetine was purchased from Sigma-Aldrich (Steinheim, Germany). Tumor necrosis factor-α (TNF-α) was purchased from Sino Biological Inc. (Peking, China). Quanti-Blue was purchased from Invitrogen and prepared according to the instructions of the manufacture.

### Cell cultures of T98G brain cells

T98G brain cells were obtained from the German Cancer Research Center (DKFZ, Heidelberg, Germany). The original source of this cell line is the American Type Culture Collection (ATCC® number: CRL-1690™). T98G cells were cultured under standard conditions (37°C, 5% CO_2_) in DMEM medium supplemented with 10% fetal bovine serum and 1% penicillin/streptomycin. Cells were passaged twice a week. All experiments were performed with logarithmically growing cells.

### Cell viability assay

Cell viability was evaluated by the resazurin assay. One hundred microliters of cell suspension were sowed into 96-well plates 1 day before the treatment with different concentrations of FAEW and fluoxetine. After 72 h, 20 μl resazurin (Sigma-Aldrich, Germany) 0.01% w/v in ddH_2_O was added to each well and the plates were incubated at 37°C for 4 h. The fluorescence was measured with an Infinite M200 Proplate Reader (Tecan, Crailsheim, Germany) using an excitation wavelength of 544 nm and an emission wavelength of 590 nm. The cytotoxic effect of the treatment was determined as a percentage compared with the untreated cells after reducing the background value caused by the medium.

### NF-κB reporter assay

HEK293 cells stably expressing the HEK-Blue-Null vector and secreted embryonic alkaline phosphatase (SEAP) reporter gene on NF-κB promoter were purchased from Invitrogen. The cells were cultured according to the recommendations from the company and passaged twice per week. The cells were treated with different concentrations of FAEW (60, 200, 600, and 2000 μg/ml) and fluoxetine (2, 6, and 20 μM) for 24, 48, or 72 h followed by TNF-α induction for 3 h. MG-132 was used as a positive control with treatment for only 1 h. The fluorescence was measured with an Infinite M200 Proplate Reader (Tecan, Crailsheim, Germany) with a wavelength of 630 nm. Background noise caused by the culture medium was subtracted from all wells. The inhibition effect of FAEW and fluoxetine toward NF-κB activity were calculated by comparison with untreated control on the basis of TNF-α induction.

### Western blotting

T98G cells were treated with FAEW and fluoxetine for 24, 48, and 72 h, MG-132 for 1 h, followed by TNF-α induction for 3 h. MG-132 was used as positive control drug. Nuclear protein extracts were prepared according to the NE-PER nuclear and cytoplasmic extraction reagent (Thermo Scientific, USA) supplemented with EDTA-free Halt Protease Inhibitor Cocktail (Thermo Scientific). Protein concentrations were measured with Nano drop 1000 spectrophotometry (Thermo Scientific). The densities of the protein bands were quantified by FluorChemQ software (Biozym Scientific Company, Oldendorf, Germany). Nuclear p65 levels were determined with an anti-NF-κB monoclonal antibody (1:3000, Cell signaling). Histone H3 protein levels served as the internal control, using the anti-Histone H3 monoclonal antibody (1:3000, Cell Signaling). The inhibition effects of FAEW and fluoxetine toward p65 protein expression were calculated by comparison with untreated TNF-α induction group after using the control group without TNF-α for the quantification of western blots.

### Molecular docking

**Figure 1:**
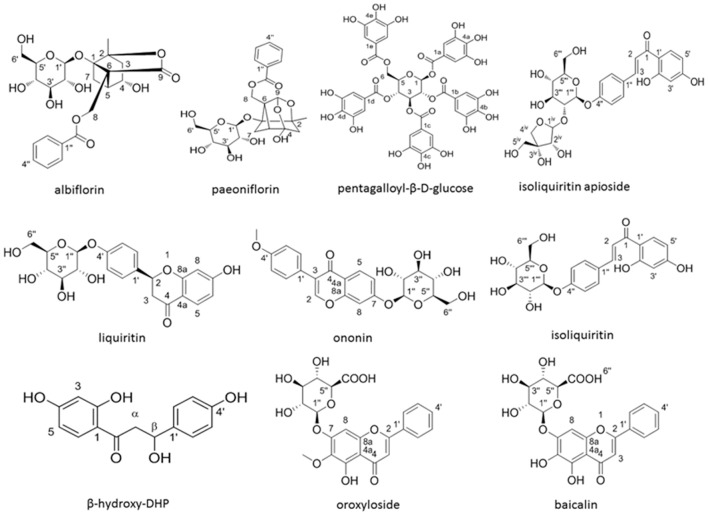
**The structures of 10 compounds isolated from FAEW**.

The ligands were selected according to the compounds in the FAEW extract identified by us using 2D-NMR- and HPLC-MS techniques (Figure 1). Together with the anti-depressant drug fluoxetine and MG-132, 12 ligands were evaluated for their binding energies to different proteins of the NF-κB pathway predicted *in silico*. PDB files of proteins were downloaded from RCSB Protein Data Bank (http://www.rcsb.org/pdb/home/home.do). The defined residues were chosen from the previous work in our laboratory (Kadioglu et al., 2016). The known inhibitor for IκK, MG-132 (PubChem ID: 462382) was selected as a standard to compare its binding modes with other compounds (Wang et al., 2013). 3D structures of these compounds were downloaded from PubChem, ChemSpider was used to convert mol files to pdb file after checking absolute and relative configuration. Molecular docking was then carried out with Auto-Dock 4.2 (The Scripps Research Institute, La Jolla, CA) following a protocol previously reported by us (Qiaoli Zhao et al., 2013). VMD (Visual Molecular Dynamics) was used for visualization of the binding modes obtained from docking. The average of the lowest binding energy of three runs was taken into account.

### Statistical analysis

*T*-test was used to calculate the significance with SPSS statistics 22, i.e., *p*-values between treatment groups and control group of three independent experiments are shown (^*^p < 0.05, ^**^p < 0.01).

### Literature review

The PubMed database was searched with the corresponding compound name as key word. Identified literature, which were related to the pharmacological activities of the compound, was classified into *in vitro, in vivo* or clinical trial reports to give a retrospective summary of the current state of knowledge. To search the published studies of the compounds from FAEW on NF-κB, NF-κB and the corresponding compound name were used as the key words.

## Results

### Canonical pathways, diseases and functions, and networks

**Figure 2:**
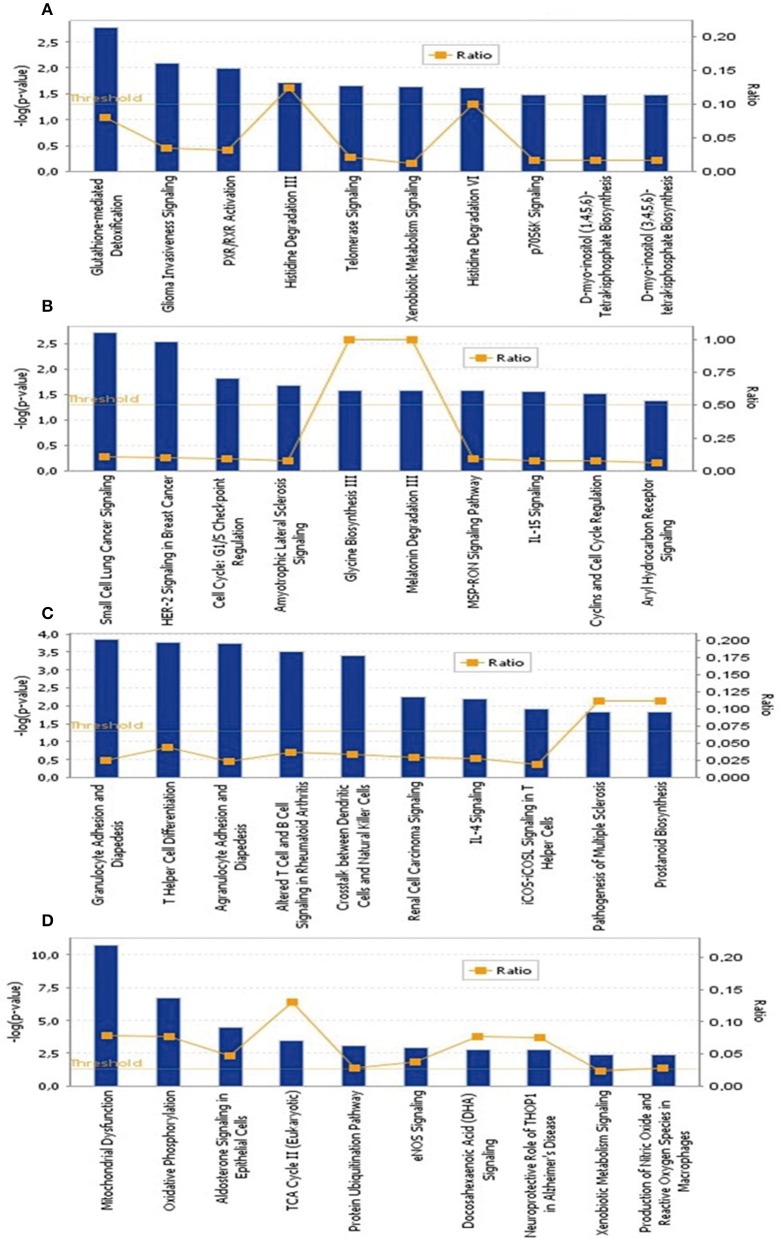
**The top 10 canonical pathways significantly affected by PTSD. (A)** Blood-based gene-expression according to [15]; **(B)** Peripheral blood mononuclear cell gene expression profiles according to [16]; **(C)** Monocyte gene expression profiles according to [13]; **(D)** *Post mortem* brain biopsy gene expression profiles according to [14].

**Figure 3:**
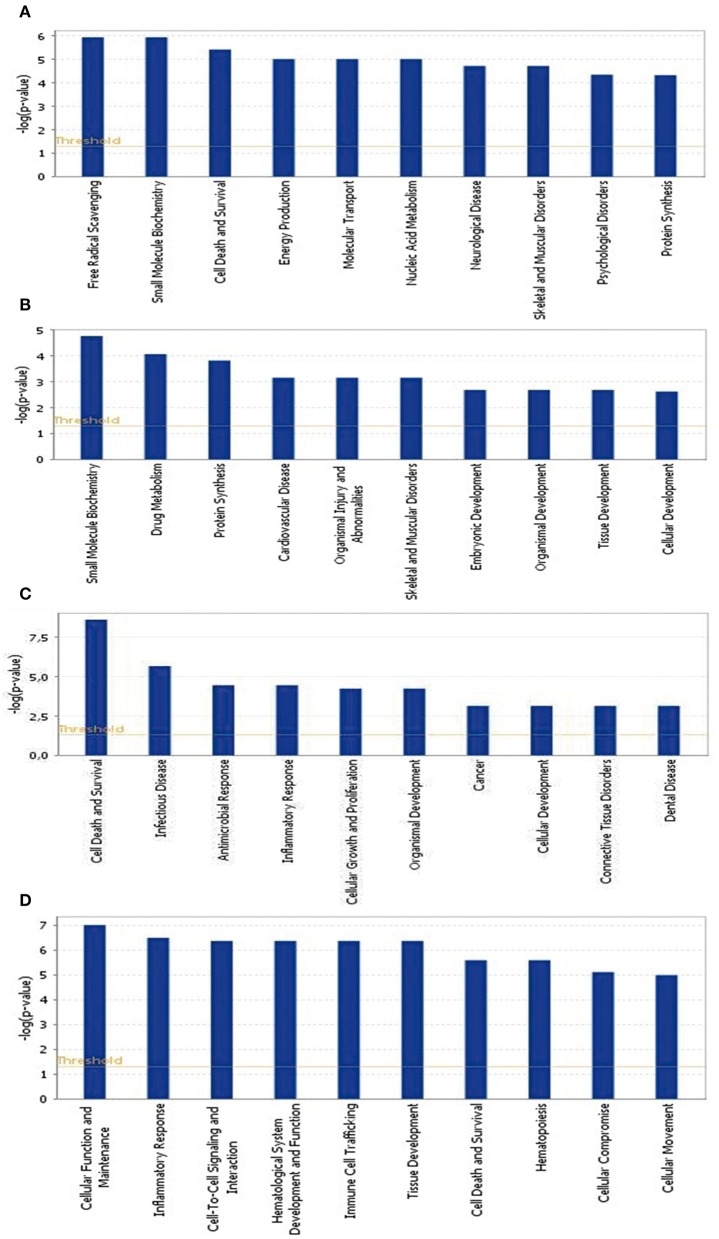
**Top 10 diseases and functions significantly affected by PTSD. (A)** Blood-based gene-expression according to [15]; **(B)** Peripheral blood mononuclear cell gene expression profiles according to [16]; **(C)** Monocyte gene expression profiles according to [13]; **(D)** *Post mortem* brain biopsy gene expression profiles according to [14].

**Figure 4:**
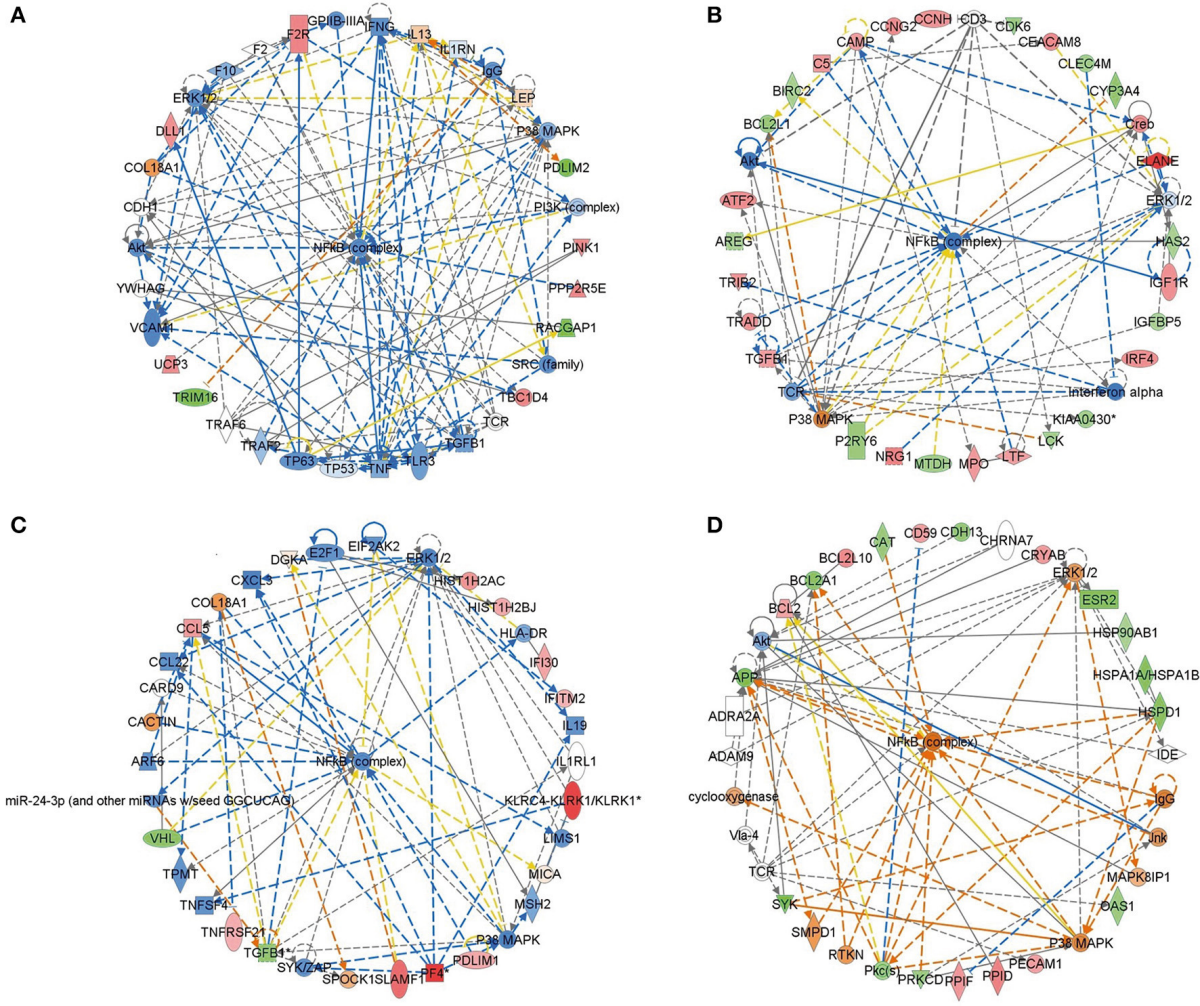
**Deregulated genes among PTSD patients**. Red colored genes were up-regulated, green colored ones were down-regulated. The arrows indicted effects of deregulated genes on other genes. Continuous lines show direct interactions, dotted lines indirect interactions. **(A)** Blood-based gene-expression according to [15]; **(B)** Peripheral blood mononuclear cell gene expression profiles according to [16]; **(C)** Monocyte gene expression profiles according to [13]; **(D)** *Post mortem* brain biopsy gene expression profiles according to [14].

To identify the common pathways related to PTSD, the top 10 canonical pathways of each of the four datasets were displayed by means of IPA analyses (Figure 2). In datasets 1 and 2, the gene profiles were involved with the cancer and tumor pathway. In dataset 3, cellular pathways “change of immune system” and “monocyte among the immune system” were significantly related. In dataset 4, the main pathways occurred in mitochondria, such as “mitochondrial dysfunction” and “oxidative phosphorylation.” Figure 3 shows the top 10 diseases and functions related to the dysregulated genes. Cellular dysfunctions, inflammation and free radical scavenging were highly involved. Cardiovascular diseases, cancer, organismal development and neurological diseases were also predicted by the dysregulated genes. Network analyses were performed to investigate the relationship between the dysregulated genes (Figure 4). Among the four datasets, NF-κB played a central role in all networks of dysregulated genes.

### Analysis of binding motifs for transcription factors in gene promoters

**Figure 5:**
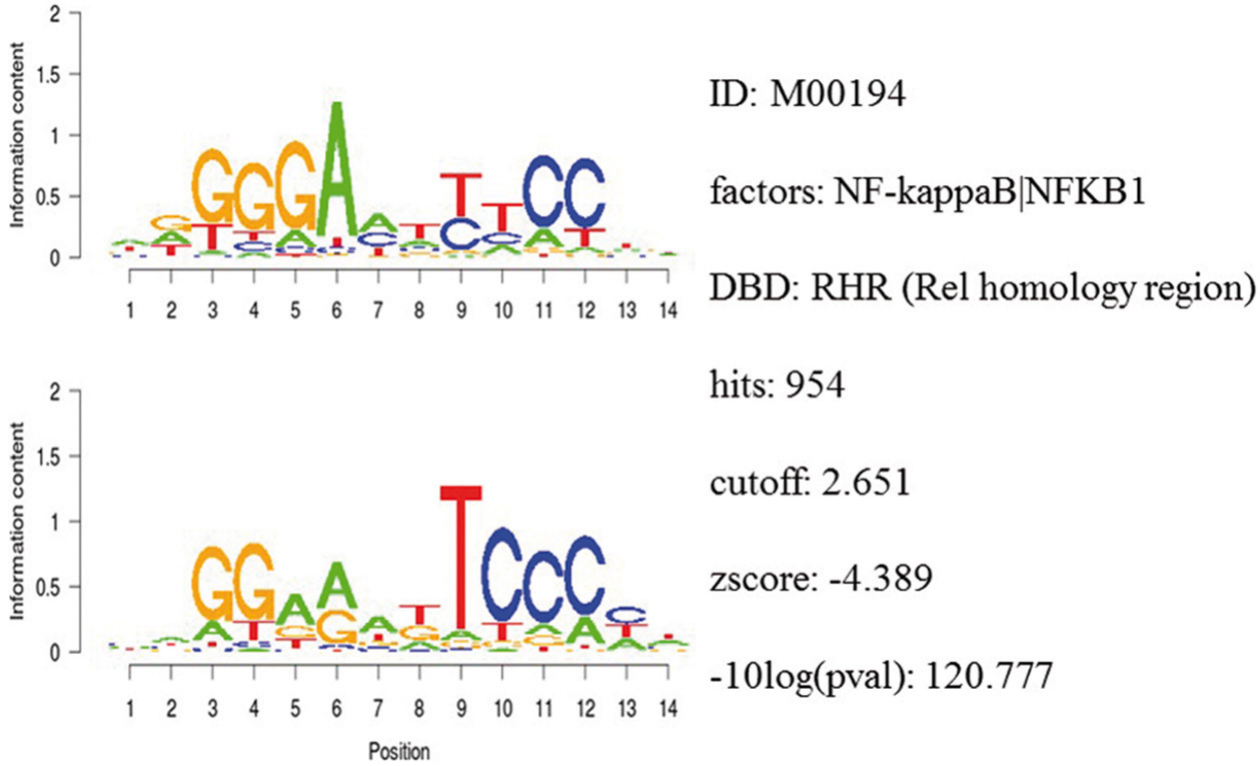
**Detailed information of the NF-κB binding motif in gene promoter sequences**.

Integrative gene promoter analyses were performed to investigate common binding motifs for transcription factors in the promoter sequences of dysregulated genes among the datasets. Table 1 shows the top ranked transcription factors. Among them was the NF-κB binding motif with a z-score of −4.39. Figure 5 shows detailed information for NF-κB.

**Table 1:** **The most pronounced gene promoter binding motifs depending on the integrative analysis of Galaxy/Cistrome**.

**ID**	**Factor**	**Hits**	**Cutoff**	**Zscore**	**−10×log(*p*-value)**
1	IKZF2|Ikzf2	33	8.733	−5.25	163.693
2	CAT8	21	8.035	−5.16	159.248
3	CCDC16	70	6.592	−5.09	155.157
4	HOXA1	285	5.666	−4.94	147.672
5	HOXD10|Hoxd10	175	7.493	−4.90	145.427
6	Meox1	235	6.41	−4.87	144.123
7	E2F1::TFDP1	569	4.626	−4.85	143.061
8	Sfpi1	179	6.952	−4.72	136.281
9	Mox1|MEOX1|CD200|NOX1	245	6.333	−4.66	133.664
10	CBFA2T2	233	4.945	−4.65	133.291
11	DAL81	72	4.577	−4.61	130.914
12	Etv1	137	6.035	−4.60	130.538
13	Hoxa1	267	5.713	−4.57	129.11
14	FOXP4	211	5.907	−4.55	128.263
15	Nkx1-1	104	6.226	−4.54	127.587
16	lhx6.1|LHX6	499	4.368	−4.43	122.535
17	MET4	340	5.212	−4.41	121.631
**18**	**NF-κB |NFKB1**	**954**	**2.651**	−**4.39**	**120.777**
19	ACE2	537	6.774	−4.25	114.451
20	Muscle TATA box	1242	1.144	−4.23	113.4

*Bold values are possible transcriptional motif related to NF-κB shown in the networks analysis*.

### Cytotoxicity of FAEW

**Figure 6:**
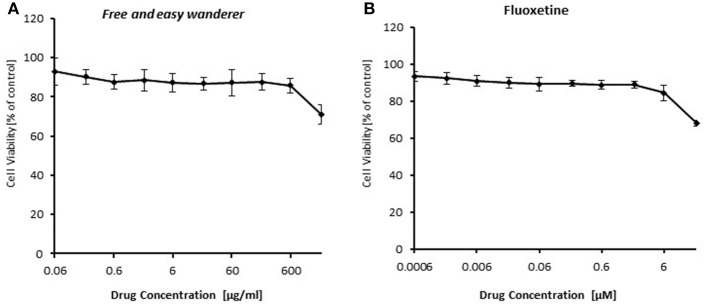
**Cytotoxicity of FAEW (A)** and fluoxetine **(B)** as determined by the resazurin assay. Shown are mean values ± SD of three independent experiments.

To identify the role of NF-κB in the acting model of FAEW, firstly, resazurin assays in HEK293 cells were performed to investigate, whether or not FAEW reveals cytotoxic effects. As shown in Figure 6, FAEW was indeed not cytotoxic at concentrations up to 2000 μg/ml. For comparison, fluoxetine was non-toxic up to 6 μM and inhibited HEK293 cells at higher concentrations.

### Inhibition of NF-κB by FAEW

**Figure 7:**
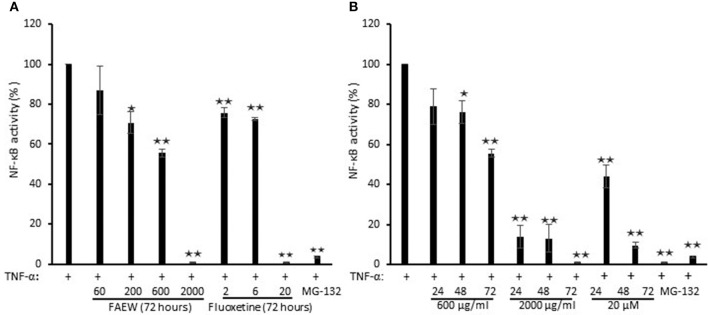
**Inhibition of NF-κB activity by FAEW and fluoxetine in HEK293 reporter cells. (A)** Concentration kinetics and **(B)** time kinetics shown are mean values ± SD of three independent experiments. ^*^p < 0.05, ^**^p < 0.01. The inhibition effects of FAEW and fluoxetine toward NF-κB activity were calculated by comparison with untreated TNF-α induction group.

To investigate whether FAEW affects PTSD through NF-κB-mediated inflammatory effects, NF-κB reporter cell assays were performed. As shown in Figure 7, FAEW showed a dose-dependent inhibition and significantly inhibited NF-κB activity at higher concentrations. In addition, the inhibitory effect strengthened in a time-dependent manner from 24 to 72 h, reaching the similar inhibitory level caused by MG-132. Fluoxetine revealed the same trend as FAEW.

### Inhibition of P65 expression by FAEW

**Figure 8:**
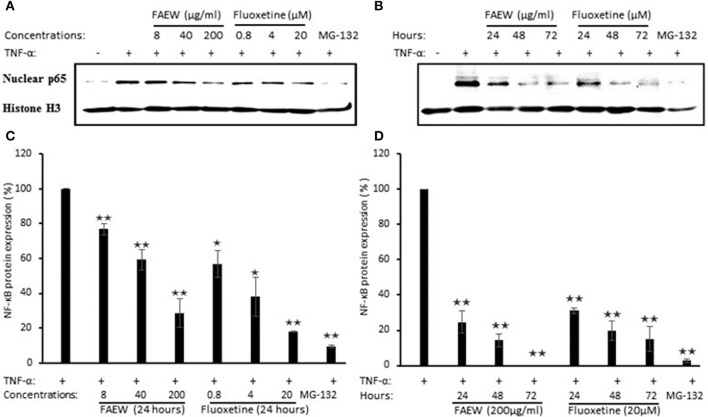
**Inhibition of p65 expression by FAEW and fluoxetine in T98G brain cells. (A)** Concentration kinetics and **(B)** time kinetics. Histone was used as loading control. **(C,D)** show the quantification of western blots shown in **(A,B)**, respectively. The inhibition effects of FAEW and fluoxetine toward p65 protein expression were calculated by comparison with untreated TNF-α induction group after using the control group without TNF-α for the quantification of western blots. Shown are mean values ± SD of three independent experiments. ^*^p < 0.05, ^**^p < 0.01.

To further confirm the inhibition of NF-κB, western blot assays were performed to investigate the role of FAEW on p65 protein expression in T98G brain cells. According to the cytotoxic assays in Figure 8, three non-cytotoxic concentrations (200, 40, and 8 μg/ml) were selected. As shown in Figures 8A,C, with treatment for 24 h, both FAEW and fluoxetine inhibited p65 expression in a dose-dependent manner. Fixed concentrations of 200 μg/ml FAEW or 20 μM fluoxetine inhibited p65 expression in a time-dependent manner from 24 to 72 h (Figures 8B,D).

### Molecular docking of compounds from FAEW to the proteins of the NF-κB pathway

**Figure 9:**
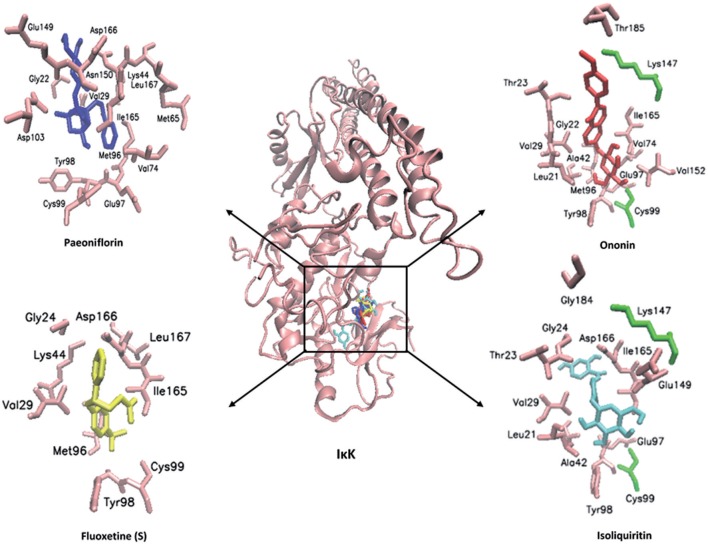
**Visualization of molecular docking of chemical compounds isolated from FAEW to IκK**.

**Figure 10:**
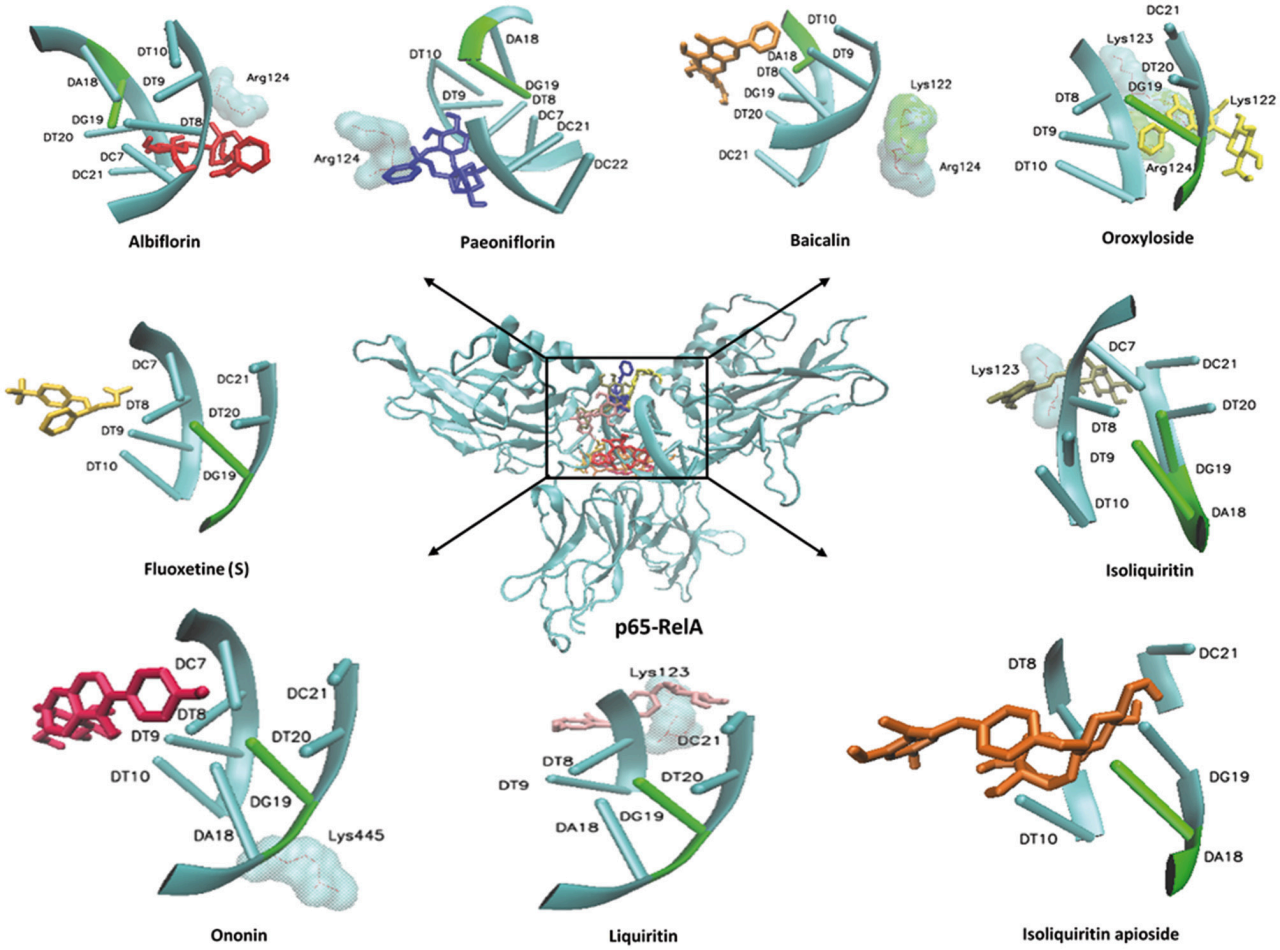
**Visualization of molecular docking of chemical compounds isolated from FAEW to p65-RelA**.

In order to explore possible interactions of compounds known to be in FAEW with the proteins of the NF-κB pathway, *in silico* molecular docking analysis were performed with 10 compounds (shown in Figure 1) in the remedy to IκK-NEMO, IκK and p65-RelA. As shown in Tables 2, 3, paeoniflorin and ononin bound to IκK and p65-RelA with low binding energies, which were comparable to the binding of MG-132. In addition, isoliquiritin apioside, albiflorin, baicalin, isoliquiritin, liquiritin and oroxyside mainly bound to p65-RelA through DNA and ATP binding sites (Figures 9, 10). The control fluoxetine (S) bound to p65-RelA with high affinity.

**Table 2:** **Binding energies of molecular docking of chemical compounds of FAEW to proteins of the NF-κB pathway**.

**Ligand**	**IκK-NEMO**		**IκK**		**p65-Rel A**	
	**Lowest binding energy (kcal/mol)**	**pKi (μM)**	**Lowest binding energy (kcal/mol)**	**pKi (μM)**	**Lowest binding energy (kcal/mol)**	**pKi (nM)**
Baicalin	−3.79 ± 0.50	1690 ± 155.56	−5.97 ± 0.04	42.47 ± 2.43	−9.68 ± 0.09	79.88 ± 11.99
β-hydroxy-DHP	−5.04 ± 0.05	203.52 ± 18.09	−5.87 ± 0.18	52.24 ± 14.46	−8.54 ± 0.04	553.70 ± 33.52
Isoliquiritin	−3.59 ± 0.17	2390.00 ± 692.97	−7.04 ± 0.08	6.92 ± 0.98	−9.97 ± 0.22	61.00 ± 4.24
Isoliquiritin apioside	−1.20 ± 0.12	132.78 ± 28.65	−6.90 ± 0.15	9.00 ± 2.28	−10.10 ± 0.74	56.59 ± 8.56
Liquiritin	−4.12 ± 0.28	1155.00 ± 247.49	−6.10 ± 0.19	34.91 ± 11.08	−9.20 ± 0.04	180.31 ± 14.99
Ononin	−3.82 ± 0.02	1600.00 ± 70.71	−7.33 ± 0.01	4.24 ± 0.07	−11.55 ± 0.08	3.43 ± 0.50
Oroxyloside	−3.32 ± 0.08	3690.00 ± 551.54	−6.16 ± 0.04	30.84 ± 1.90	−9.41 ± 0.02	127.50 ± 4.95
Fluoxetine (R)	−4.35 ± 0.05	652.97 ± 54.98	−5.61 ± 0.06	77.65 ± 8.25	−7.35 ± 0.07	4093.33 ± 475.01
Fluoxetine (S)	−4.14 ± 0.05	928.59 ± 79.41	−5.90 ± 0.03	47.15 ± 2.48	−7.63 ± 0.03	2543.33 ± 123.42
Albiflorin	−5.05 ± 0.30	211.37 ± 52.00	−6.63 ± 0.16	14.23 ± 3.77	−10.66 ± 0.50	23.85 ± 3.12
Paeonflorin	−4.41 ± 0.04	591.13 ± 29.66	−7.83 ± 0.21	2.09 ± 0.27	−11.25 ± 0.03	5.45 ± 0.07
Pentagalloyl-β-D-glucose	−0.67 ± 0.01	320580.00 ± 150.00	−2.39 ± 0.49	19090.00 ± 3490.00	−6.09 ± 0.21	35.50 ± 1.27
MG-132	−3.50 ± 0.63	47053.00 ± 1090.00	−7.75 ± 0.35	2.31 ± 0.33	−9.07 ± 0.29	172.10 ± 27.01

*IκK, IκB kinase; IκK, NEMO. NF-κB essential modulator; NF-κB, nuclear factor kappa; light, chain-enhancer of activated B cells*.

**Table 3:** **Hydrogen bonds and amino acid residues identified by molecular docking of chemical compounds of FAEW to proteins of the NF-κB pathway**.

**Protein**	**Ligand**	**Residues making H bonds**	**Residues involved in hydrophobic interactions**
IκK	Baicalin		Thr23, Val29, Ala42, Lys44, Met65, Met96, Glu97, Tyr98, Cys99, Asp103, Val152, Ile165, Asp166, Leu167
	β-hydroxy-DHP	Lys44	Thr23, Val29, Ala42, Lys44, Met96, Glu97, Ile165, Asp166, Leu167
	Isoliquiritin	Cys99, Lys147	Leu21, Thr23, Gly24, Val29, Ala42, Glu97, Tyr98, Cys99, Lys147, Glu149, Ile165, Asp166, Gly184
	Isoliquiritin apioside	Lys44, Cys99	Thr23, Gly24, Val29, Ala42, Lys44, Met96, Glu97, Tyr98, Cys99, Lys147, Glu149, Ile165, Asp166, Leu167, Gly184
	Liquiritin	Lys147	Leu21, Thr23, Gly24, Ala42, Glu97, Cys99, Lys147, Glu149, Val152, Ile165, Asp166
	Ononin	Cys99, Lys147	Leu21, Gly22, Thr23, Val29, Ala42, Val74, Met96, Glu97, Tyr98, Cys99, Lys147, Val152, Ile165, Thr185
	Oroxyloside	Cys99	Thr23, Val29, Ala42, Lys44, Met65, Met96, Glu97, Tyr98, Cys99, Asp103, Glu149, Val152, Ile165, Asp166, Leu167
	Fluoxetine (R)		Gly24, Val29, Ala42, Lys44, Met65, Met96, Tyr98, Ile165, Asp166, Leu167
	Fluoxetine (S)		Gly24, Val29, Lys44, Met96, Tyr98, Cys99, Ile165, Asp166, Leu167
	Albiflorin	Cys99	Gly22, Val29, Lys44, Met 65, Val74, Met96, Glu97, Tyr98, Cys99, Asp103, Glu149, Asn150, Ile165, Asp166, Leu167
	Paeoniflorin		Gly22, Val29, Lys44, Met65, Val74, Met96, Glu97, Tyr98, Cys99, Asp103, Glu149, Asn150, Ile165, Asp166, Leu167
	Pentagalloyl-β-D-glucose	Glu100	Leu21, Gly22, Thr23, Val29, Ala42, Lys44, Glu61, Met65, Val73, Val74, Ala76, Leu94, Met96, Tyr98, Cys99, Glu100, Gly102, Asp103, Glu149, Asn150, Ile151, Val152, Ile165, Asp166, Leu167
	MG-132		Leu21, Thr23, Val29, Ala42, Met65, Val73, Val74, Met96, Glu97, Tyr98, Asp103, Val152, Ile164, Ile165, Asp166, Leu167
p65-RelA	Baicalin	DA18, Lys122	DT8, DT9, DT10, DA18, DG19, DT20, DC21, Lys122, Arg124
	β-hydroxy-DHP	DG19	DT8, DT9,DT10, DA18, DG19, DT20, DC21
	Isoliquiritin	DA18, DG19	DC7, DT8, DT9, DT10, DA18, DG19, DT20, DC21, Lys123
	Isoliquiritin apioside	DA18	DT8, DT10, DA18, DG19, DC21
	Liquiritin	DG19	DT8, DT9, DA18, DG19, DT20, DC21, Lys123
	Ononin	DG19	DC7, DT8, DT9, DT10, DA18, DG19, DT20, DC21, Lys445
	Oroxyloside	DG19, Lys122, Arg124	DT8, DT9, DT10, DG19, DT20, DC21, Lys122, Lys123, Arg124
	Fluoxetine (R)	DA18, DG19	DC7, DT8, DT9, DT10, DA18, DG19, DT20, DC21
	Fluoxetine (S)	DG19	DC7, DT8, DT9,DT10, DG19, DT20, DC21
	Albiflorin	DG19	DC7, DT8, DT9, DT10, DA18, DG19, DT20, DC21, Arg124
	Paeoniflorin	DG19	DC7,DT8, DT9, DT10, DA18, DG19, DC21, DC22, Arg124
	Pentagalloyl-β-D-glucose	DG19	DC7, DC8, DT9, DG19, DT20, DC21, DC22, Lys123, Arg124
	MG-132	DG19	DA6, DC7, DT8, DT9, DT10, DA18, DG19, DT20, DC21, DC22, Lys123

### Literature review on the pharmacological activity of the compounds from FAEW

To further characterize the therapeutic potential of the candidate compounds investigated by molecular docking, we performed a literature review of the current research on the isolated compounds of FAEW. As shown in Table 4, paeoniflorin, albiflorin and baicalin were reported to act *in vitro* against inflammation and oxidative stress, and to be effective in *vivo* against depression, and Parkinson's disease. Furthermore, clinical trials were carried out among herbal formulae in inflammation-related diseases, such as Japanese traditional recipe *Shakuyakukanzoto*, and the Chinese herbal formula *Xiongshao capsule*. *In vivo* studies on isoliquiritin and liquiritin showed their activity against depression and cognitive-related diseases, for instance, Parkinson's disease. As shown in Table 5, paeoniflorin, baicalin and liquiritin have been reported in many *in vitro* and *in vivo* studies to reduce inflammatory processes realted to numerous diseases through inhibiting NF-κB, which implies their role as constituents of FAEW acting against inflammatory reactions related to PTSD.

**Table 4:** **Literature review on the pharmacological activity of the compounds from FAEW**.

**Compounds**	**Pharmacological activity**		
	***In vitro***	***In vivo***	**Clinical trial**
Paeoniflorin	Anti-inflammation and oxidative stress (Dong et al., 2015); neuroprotection (Mao et al., 2012); apoptosis (Yang et al., 2016a)	Parkinson's disease (Gu et al., 2016); depression (Huang et al., 2015); neuropathic pain (Zhou et al., 2016a); non-alcoholic steatohepatitis (Ma et al., 2016)	Rheumatoid arthritis (Chen et al., 2013); muscle cramps and abdominal pains (Sadakane et al., 2015); restenosis after percutaneous coronary intervention (Shang et al., 2011)
Albiflorin	Oxidative stress (Suh et al., 2013); neuroprotection (Kim et al., 2009)	Anti-depression (Song et al., 2015); Parkinson's disease (Ho et al., 2015); neuropathic pain (Zhou et al., 2016a; Zhang et al., 2016b)	Muscle cramps and abdominal pains (Sadakane et al., 2015)
Baicalin	Inflammation (Min et al., 2015); apoptosis (Li et al., 2015b); oxidative stress (Zheng et al., 2014)	Alcoholic liver injury (Wang et al., 2016b); depression/anxiety (Zhang et al., 2016a); renal damage (Zhang et al., 2016d); memory impairment (Wang et al., 2016c)	Ulcerative colitis (Yu et al., 2015)
Isoliquiritin	Anti-allergic activity (Kaur et al., 2009)	Depression (Wang et al., 2008); antifungal activity (Luo et al., 2016)	Depression (Su et al., 2014)
Liquiritin	Neuroprotection (Teng et al., 2014)	Cognitive deficits (Jia et al., 2016); Parkinson's disease (Huang et al., 2016); depression (Farahani et al., 2015); focal cerebral ischemia (Sun et al., 2010)	Melisma (Amer and Metwalli, 2000)
β-hydroxy-DHP	Apoptosis (Rafi et al., 2002)		
Isoliquiritin apioside	Anti-genotoxic (Kaur et al., 2009)		
Oroxyloside		Colitis (Wang et al., 2016d); inflammation-related diseases (Fong et al., 2015)	

**Table 5:** **Literature review of the effect of chemical constituents of FAEW against NF-κB**.

**Compounds**	**Pharmacological activity on NF-κB**	
	***In vitro***	***In vivo***
Paeoniflorin	Parkinson's disease (Liu et al., 2016a); immunomodulation (Zhai et al., 2016); Alzheimer's disease (Liu et al., 2015); apoptosis (Dong et al., 2015); morphine tolerance (Jiang et al., 2015); anti-inflammation and immunomodulation (Gan et al., 2014); sepsis (Zhang et al., 2014b); obesity (Kong et al., 2013); cardiac remodeling (Zhou et al., 2013)	Renal function (Liu et al., 2016c); non-alcoholic steatohepatitis(Ma et al., 2016); cardiac dysfunction (Zhai and Guo, 2016); vascular dementia (Zhang et al., 2015b); Alzheimer's Disease (Zhang et al., 2015a); arthritis (Yi, 2014); hepatitis (Chen et al., 2015); colitis (Zhang et al., 2014a); learning dysfunction and brain damage (Guo et al., 2012); lung injury (Zhou et al., 2011)
Baicalin	Myofibroblast differentiation (Shin et al., 2016); atherosclerosis (Wang et al., 2016a); haemophilus parasuis infection (Fu et al., 2016); mastitis (Guo et al., 2013); apoptosis (Yang et al., 2016b)	Asthma (Liu et al., 2016b); periodontitis (Sun et al., 2016); allergic diseases (Zhou et al., 2016b); arthritis (Wang et al., 2014); brain edema (Zhou et al., 2014); lung injury (Ding et al., 2016); liver injury (Wan et al., 2008); ischemic stroke (Singh and Chopra, 2014); cerebral ischemia (Tu et al., 2011)
Isoliquiritin	Inflammatory responses (Kim et al., 2008)	
Liquiritin	Endothelial dysfunction (Zhang et al., 2013)	Myocardial fibrosis (Zhang et al., 2016c); acute Lung Injury (Tao et al., 2016)
Oroxyloside		Colitis (Wang et al., 2016d)

## Discussion

Posttraumatic stress disorder (PTSD) is a delayed and lasting psychological stress disorder after traumatic events. It was initially diagnosed among veterans of the Vietnam War. Subsequent studies indicated that victims of disasters were also potentially suffering from PTSD (Cavanagh et al., 2014). It has been calculated that life time prevalence of PTSD in adults is 7.8%, while women have higher risk than men (20.4 vs. 8.2%) despite experiencing fewer traumas (Kessler et al., 2005; Ditlevsen and Elklit, 2012). Pre-trauma factors, such as lower socioeconomic status, parental neglect and poor social support increase the risk (Hong and Efferth, 2015). In addition, more and more studies reported the correlation between PTSD and other physiological diseases, such as diabetes (Agyemang et al., 2012), cancer (Abbey et al., 2015) and cardiovascular diseases (Boscarino, 2008), or psychiatric diseases, such as depression and anxiety (Farr et al., 2014). In our studies, we analyzed the microarray-based transcriptome-wide mRNA expression profiling of patients with PTSD. As shown in the present investigation, the top 10 pathways and functions, dysregulated genes of PTSD patients were related to oxidative stress and inflammatory response, and involved with a wide array of psychological or physiological diseases, which might be taken as a general hint for common pathogenic factors between PTSD and metabolic syndrome, cardiovascular diseases, cancer, as well as psychological diseases, e.g., depression, suicidality and anxiety, implying that a holistic way to investigate PTSD might be one approach to achieve multiple-targets.

Increasing evidence indicated an involvement of immune system in fear- and anxiety-based disorders. Recent studies suggested that inflammation is associated with increased basal ganglia glutamate in patients in depression (Haroon et al., 2016). Furthermore, inflammasome signaling affects anxiety- and depressive-like behavior and gut microbiome composition, and suggesting that the gut microbiota-inflammasome-brain axis could be novel therapeutic targets for psychiatric disorders (Wong et al., 2016). In our studies, network analyses revealed that NF-κB was activated in both the peripheral and central nervous system. In addition, promoter binding motif search of genes revealed that NF-κB was among the most important transcription factors. These results indicated that NF-κB may be an important immunological component of inflammatory processes in PTSD. Therefore, we hypothesized a link between the therapeutic effect of FAEW on PTSD, and NF-κB as relevant underlying mechanism.

*Free and Easy Wanderer* (FAEW) is a poly-herbal preparation, which is widely used in Chinese clinics for the treatment of depression, premenstrual dysphoric disorder, climacteric syndrome, and Parkinson's disease induced by antipsychotic drugs. An *in vivo* study indicated that FAEW ameliorated PTSD-like behavior and cognitive impairments in stressed rats (Wang et al., 2009). Subsequent clinical studies also reported good efficacy, safety, and tolerability in post-stroke depression patients (Li et al., 2008). In our studies, we observed that FAEW had a wide safety range and showed a dose and time-dependent inhibition of NF-κB activity in HEK293 cells as well as of protein expression of NF-κB in T98G brain cells. Hence, FAEW might exert anti-anxiety effects through NF-κB-mediated anti-inflammatory process. Among a panel of 10 compounds, paeoniflorin, isoliquiritin, isoliquiritin apioside, and ononin exerted high affinity to IκK and p65-RelA. Albiflorin, baicalin, liquiritin, and oroxyloside were predicted to strongly bind to p65-RelA. Apart from lipophilic interactions, which widely occur among the interactions between lipophilic groups of molecules and the nonpolar side-chains of residues, such as Ile, Leu, Val, and Phe, hydrogen bonds are the main interaction force to promote molecules bind to proteins. For example, paeoniflorin exerted strong affinity with IκK and p65 through multiple hydrogen bonds involving the OH groups of its glucose moiety. The hemiacetal OH-group in the core (position 5) and the 3′- and 4′-OH of paeoniflorin were predicted to bind to IκK residues Thr23, Cys99 and Glu 97, respectively. In the interaction of the same compound with p65, the 5-OH and the glucose OH groups in positions 3′, 4′, and 6′ bound to the p65 residues DT9, DC22, DT8, and DC21, respectively. Besides, p65 residue DG19 donates a hydrogen bond to the benzoate carbonyl of paeoniflorin. The same type of interactions for the other compounds from FAEW can be applied to explain their activities toward NFκB, such as ononin, isoliquiritin. Furthermore, paeoniflorin, albiflorin, baicalin, isoliquiritin, and liquiritin were reported to be active against depression and Parkinson's disease in *in vivo* studies and clinical trials, and paeoniflorin, baicalin and liquiritin were reported to inhibit NF-κB *in vitro* and *in vivo*. These data demonstrate that FAEW is constituted by a wide array of diverse anti-depressant natural drugs with strong anti-inflammatory activity. Importantly, some compounds have been indeed demonstrated to pass the blood-brain barrier and reach brain tissue, e.g., albiflorin, paeoniflorin, liquiritin, which may might explain the effect of FAEW in the central nervous system (Li et al., 2015a). In addition, it is also quite interesting to observe that some compounds were used to treat colitis *in vivo* and in clinic studies, which might imply their participation in the balance of the gut microbiota-inflammasome-brain axis (Yu et al., 2015; Wang et al., 2016d).

Fluoxetine first emerged in the 1970s as selective serotonin uptake inhibitor for the treatment of depression due to its safer profile, fewer side effects, and greater tolerability than former drugs (Wilde and Benfield, 1998). Subsequent studies indicated that it also reveals strong anti-inflammatory properties, and its effect on NF-κB was recognized recently (Mackay et al., 2009; Cui et al., 2016). In addition to depression, clinical studies demonstrated that fluoxetine also significantly improved symptom clusters of PTSD (Martenyi and Soldatenkova, 2006). Therefore, in our study, fluoxetine was selected as standard control drug, and it was proved that fluoxetine decreased NF-κB levels, which contributes to the explanation of its effects in the treatment of PTSD.

Different from Western medicine with one drug- one target action model, TCM pursues a multi-target model in different organs and tissues of the body and achieved a lot in the application of acupuncture worldwide. Although Chinese herb formulae are still a matter of discussion, their clinical effects in Asian countries have a high reputation. In our studies, we observed that, with diverse active compounds, FAEW exerted strong anti-inflammatory effects toward NF-κB, which were comparable to the antidepressant drug, fluoxetine. It is safe and approachable for the treatment of inflammation-related diseases. A limitation of this investigation represents the lack of *in vivo* PTSD models to validate the effect of FAEW in more detail and investigate possible clinical effects. The literature review provided us some hints for the future studies, to confirm and investigate the activity of different components of FAEW. Future studies will focus on the performance of *in vivo* validation and clinical trials to further evaluate the therapeutic potential of FAEW to treat PTSD-related diseases.

## Author contributions

TE came up the idea and designed the experiment. CH conducted the biological experiments. JC and CW assisted the bioinformatic stuff. ET and TO were responsible for the isolation and identification of the compounds, respectively. AS and UK performed the studies, accordingly.

### Conflict of interest statement

The authors declare that the research was conducted in the absence of any commercial or financial relationships that could be construed as a potential conflict of interest.

## Abbreviations

FAEW: *Free and Easy Wanderer*
HPLC: high pressure liquid chromatography
IκK: inhibitor kappa B kinase
IPA: ingenuity pathway analysis
MS: mass spectrometry
NF-κB: nuclear factor “kappa light chain enhancer” of activated B-cells
NMR: nuclear magnetic resonance-mass spectrometry
PTSD: posttraumatic stress disorder
TCM: traditional Chinese medicine.
